# Carbetocin versus oxytocin during elective cesarean delivery: A randomized double-blind trial

**DOI:** 10.12669/pjms.42.9.13879

**Published:** 2026-09

**Authors:** Baran Tureli, Nurettin Kurt, Erbil Karaman, Nureddin Yuzkat

**Affiliations:** 1 Baran Tureli Dept. of Anesthesiology & Reanimation, Health Science University, Van Training and Research Hospital, Van, Turkey; 2 Nurettin Kurt Department of Anesthesiology and Reanimation, Faculty of Medicine, Van Yuzuncu Yil University, Van, Turkey; 3 Erbil Karaman Department of Obstetrics and Gynecology, Faculty of Medicine, Van Yuzuncu Yil University, Van, Turkey; 4 Nureddin Yuzkat Department of Anesthesiology and Reanimation, Faculty of Medicine, Van Yuzuncu Yil University, Van, Turkey

**Keywords:** Cesarean delivery, Carbetocin, Intraoperative bleeding, Oxytocin, Uterotonic agents

## Abstract

**Objective:**

Postpartum hemorrhage (PPH) remains a leading cause of maternal morbidity following cesarean delivery. This study aimed to compare the effects of carbetocin and oxytocin on intraoperative blood loss during elective cesarean delivery, with secondary evaluation of uterine tone, additional uterotonic requirements, and intraoperative hemodynamic parameters.

**Methodology:**

This prospective, randomized, double-blind study was conducted at Van Yuzuncu Yil University Dursun Odabaş Medical Center between 2023 and 2024 and included 100 patients undergoing elective cesarean delivery. Patients were randomly assigned to receive either oxytocin (Group O; 0.6 IU intravenous bolus followed by a 15 IU/h infusion) or carbetocin (Group C; 100 μg administered intravenously over 30–60 seconds). Patients with major obstetric complications, placental invasion disorders, hypertensive disorders of pregnancy, significant cardiovascular or respiratory disease, or hepatic or renal dysfunction were excluded. Intraoperative blood loss, uterine tone, additional uterotonic requirements, and hemodynamic parameters were compared.

**Results:**

Baseline demographic characteristics were comparable between groups (p>0.05). Intraoperative blood loss was significantly lower in the carbetocin group than in the oxytocin group (p<0.001). The need for additional uterotonic agents was significantly lower in the carbetocin group both within the first 5 minutes (2% vs. 12%) and throughout surgery (10% vs. 26%) (p<0.05 for both). Uterine tone was significantly higher in the carbetocin group at 5 minutes (p=0.016), whereas higher scores were observed in the oxytocin group at 10 minutes (p=0.034). No clinically significant differences were observed in intraoperative hemodynamic parameters.

**Conclusion:**

Although carbetocin and oxytocin demonstrated comparable intraoperative hemodynamic profiles, carbetocin was associated with reduced intraoperative blood loss, improved early uterine tone, and a lower requirement for additional uterotonic agents. Its single-dose administration and favorable safety profile make it a practical and effective uterotonic option for elective cesarean delivery.

## INTRODUCTION

Postpartum hemorrhage (PPH) is one of the leading causes of maternal mortality and morbidity worldwide, particularly following cesarean delivery.^1^ The World Health Organization (WHO) defines PPH as blood loss exceeding 500 mL after vaginal delivery and 1000 mL after cesarean section. Among the etiological factors, uterine atony—resulting from inadequate uterine contraction—remains the most common cause of PPH. Therefore, the prophylactic use of uterotonic agents is essential for promoting uterine contractility and reducing postpartum bleeding.^2^–^4^

Oxytocin is currently the most commonly used uterotonic agent for the active management of PPH. However, its clinical effectiveness is limited by a short half-life and the need for repeated dosing despite its rapid onset of action. To overcome these limitations, carbetocin was developed as a long-acting synthetic analogue of oxytocin. Structurally similar to oxytocin, carbetocin has a prolonged duration of action, allowing effective uterine contraction with a single dose. Owing to these pharmacological properties, carbetocin has been recommended as an alternative to oxytocin, particularly for the prevention of PPH in high-risk pregnancies.^4^,^5^

Although recent studies suggest that carbetocin may be more effective than oxytocin in reducing the incidence of PPH, direct comparisons of their intraoperative uterine effects during elective cesarean sections remain limited. Furthermore, there is no consensus regarding the optimal dosage and route of administration of carbetocin. Considering the increasing global rates of cesarean delivery and the elevated risk of uterine atony-related hemorrhage, the selection of an effective and safe uterotonic agent is of paramount importance.^6^

The primary aim of this study was to compare the effects of carbetocin and oxytocin on intraoperative blood loss during elective cesarean delivery under general anesthesia. Secondary objectives were to evaluate uterine contractility, the need for additional uterotonic agents, and intraoperative hemodynamic stability.

## METHODOLOGY

The study was conducted between December 2023 to September 2024 at the Department of Anesthesiology and Reanimation, in collaboration with the Department of Obstetrics and Gynecology at Van Yuzuncu Yil University Dursun Odabaş Medical Center and included 100 patients scheduled for elective cesarean section.

### Ethical Approval:

This prospective, randomized, double-blind study was approved by the Clinical Research Ethics Committee of Van Yuzuncu Yil University (Decision no: 03; Dated: October 25, 2023, and registered at ClinicalTrials.gov (NCT06956365). Written informed consent was obtained from all participants.

### Inclusion & Exclusion Criteria:

Patients aged 18–40 years with American Society of Anesthesiologists (ASA) physical status II who underwent elective cesarean delivery under general anesthesia were included. Exclusion criteria were ASA physical status III–IV, emergency surgery, cardiac or respiratory disease, placental invasion anomalies (placenta previa, accreta, or percreta), hypertensive disorders of pregnancy (preeclampsia, eclampsia, HELLP syndrome), history of two or more previous cesarean deliveries, chronic use of medications affecting cardiovascular or hemodynamic responses, and hepatic or renal dysfunction.

These exclusion criteria were selected to reduce potential confounding factors affecting intraoperative blood loss, uterine tone, and uterotonic requirements, thereby ensuring a more homogeneous study population.

All patients were evaluated preoperatively by the Department of Anesthesiology and Reanimation at least one day before surgery. Preoperative assessment included physical examination and routine laboratory tests. Prior to surgery, a final evaluation was performed and patients were re-informed about the study and anesthesia procedures.

A total of one hundred patients were randomized into two equal groups (n=50) using the sealed envelope technique. Allocation concealment was ensured by separating the investigator administering the uterotonic agent from the investigator collecting outcome data. Demographic data (age, height, weight, body mass index, and ASA classification) were recorded.

Standard monitoring was applied upon arrival in the operating room. Following preoxygenation with 100% oxygen for three minutes, general anesthesia was induced with propofol (2 mg/kg) and rocuronium (0.6 mg/kg). Tracheal intubation was performed using a 7.0-mm cuffed endotracheal tube, and mechanical ventilation was maintained with a tidal volume of 8–10 mL/kg and a respiratory rate of 10–12 breaths/min. Anesthesia was maintained with 1% sevoflurane in 100% oxygen until delivery and with 1–2% sevoflurane in a 50% oxygen–air mixture thereafter. Intravenous fentanyl (1.5 µg/kg) was administered after delivery, and additional rocuronium (0.01 mg/kg) was given if required. A standardized general anesthesia protocol was used for all patients to minimize variability related to anesthetic technique and to ensure consistent comparison of uterotonic effects between the study groups.

Following placental delivery, patients received the assigned uterotonic agent. Group-O received oxytocin (30 IU diluted in 1000 mL 0.9% NaCl), administered as a 0.6 IU slow intravenous bolus over one minute followed by continuous infusion at 15 IU/h. The oxytocin regimen was selected based on institutional practice and previous literature supporting prophylactic oxytocin administration by slow intravenous bolus followed by continuous infusion during cesarean delivery.^7^,^8^ Group-C received carbetocin (100 µg diluted in 20 mL 0.9% NaCl), administered intravenously over 30–60 seconds. Fluid administration was standardized, and both groups received equivalent intravenous volumes during the 30-minute monitoring period.

Hemodynamic parameters (heart rate, systolic and diastolic blood pressure, mean arterial pressure, and peripheral oxygen saturation) were recorded before uterotonic administration and at 1, 5, 10, 15, 20, 25, and 30 minutes thereafter. Uterine tone was assessed at 1, 5, and 10 minutes using a 0–10 numeric rating scale. Additional uterotonic requirements and total intraoperative blood loss were recorded.

Hypotension was defined as systolic blood pressure <90 mmHg or a >20% decrease from baseline and was treated with intravenous ephedrine (5 mg boluses). Persistent mean arterial pressure <55 mmHg despite treatment was considered deep hypotension and resulted in exclusion. A total of five patients (three in the oxytocin group and two in the carbetocin group) were excluded from the final analysis because of deep hypotension, as presented in the study flow diagram. Bradycardia (heart rate <50 bpm) was treated with intravenous atropine (1 mg).

Uterine tone was evaluated by the same surgical team at 1, 5, and 10 minutes using a 0–10 numeric rating scale, where higher scores indicated better uterine contraction. Consistent assessment by the same surgical team was maintained throughout the study to reduce interobserver variability. If the tone was ≤7, uterine massage was applied. If no adequate improvement occurred, the anesthesia team was informed and 0.2 mg of methylergometrine (Metiler ampoule 0.2 mg/mL) was administered intramuscularly as an additional uterotonic. After the 10-minute assessment period, the need for additional uterotonic agents was determined according to the attending surgeon’s routine intraoperative clinical evaluation of uterine contraction and bleeding status. Total intraoperative blood loss was estimated using a combination of suction canister volume (after subtraction of irrigation fluids), surgical sponge count, and visual assessment of blood loss within the operative field. The same surgical and anesthesia teams performed all assessments according to routine institutional practice to minimize measurement variability.

### Sample size calculation:

The sample size was estimated based on previously published data comparing intraoperative blood loss between carbetocin and oxytocin during cesarean delivery. In that study, mean blood loss was 571.42±166.26 mL in the carbetocin group and 730.95±227.20 mL in the oxytocin group. Assuming a mean difference of 159.5 mL, a pooled standard deviation of approximately 199 mL, a two-sided alpha level of 0.05, and a statistical power of 80%, at least 25 patients were required in each group.^9^ To compensate for potential exclusions, protocol deviations, and incomplete data, 50 patients were enrolled in each group, resulting in a total sample size of 100 patients.

### Statistical analysis:

Descriptive statistics were expressed as mean, standard deviation, median, minimum, maximum, frequency, and percentage. Normality of distribution was assessed using the Kolmogorov–Smirnov and Shapiro–Wilk tests. For normally distributed independent quantitative variables, the independent samples t-test was used; for non-normally distributed variables, the Mann–Whitney U test was applied. Chi-square test was used for qualitative data, and Fisher’s exact test was employed when chi-square assumptions were not met. All analyses were performed using SPSS version 28.0.

## RESULTS

Of the 109 patients assessed for eligibility, one hundred who met the inclusion criteria were included in the study (Fig.1). There were no significant differences between Group-O and Group-C in terms of age, height, weight, Body Mass Index (BMI), or smoking status (p>0.05). The intraoperative blood loss was significantly lower in Group-C compared to Group-O (p<0.05) (Table-I).

**Fig.1:**
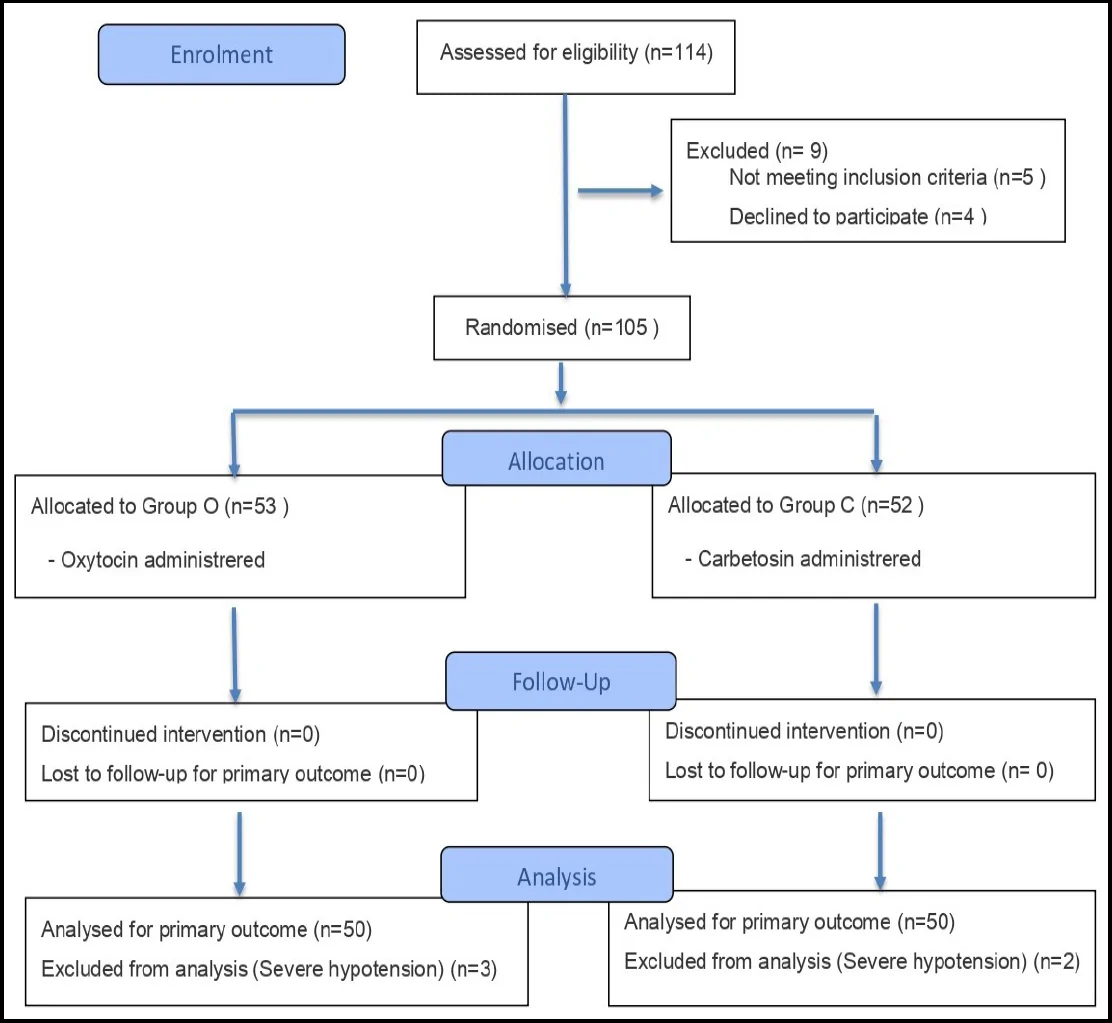
CONSORT flow diagram of patients through the study.

**Table-I:** Comparison of demographic data and intraoperative blood loss between the groups.

		Group-O (n:50)				Group-C (n:50)				p-value
		Mean±SD/n-%			Median	Mean±SD/n-%			Median	
Age (years)		30.4	±	5.3	30.0	30.3	±	5.6	30.0	0.884 ^t^
Height (cm)		164.4	±	5.9	165.0	164.2	±	4.6	164.0	0.913 ^m^
Weight (kg)		80.8	±	11.3	80.0	80.1	±	11.3	80.0	0.871 ^m^
BMI (kg/m2)		29.9	±	3.9	29.3	30.4	±	4.3	30.7	0.377 ^m^
Smoking	(-)	48		96.0%		48		96.0%		1.000 ^X²^
	(+)	2		4.0%		2		4.0%	
Intraoperative Blood loss		522.4	±	91.4	550.0	421.0	±	75.0	400.0	0.000 ^m^

^t^ Independent sample t test / ^m^ Mann-whitney u test / ^X²^ Chi-square test, BMI: body mass index.

There were no significant differences between Group-O and Group-C in systolic and diastolic blood pressure values at any measured time point (p>0.05) (Fig.2, Fig.3). The mean arterial pressure at one minute was significantly lower in Group-C compared to Group-O (p<0.05). There were no significant differences between Group-O and Group-C in mean arterial pressure at 5, 10, 15, 20, 25, and 30 minutes (p>0.05) (Fig.4).

**Fig.2:**
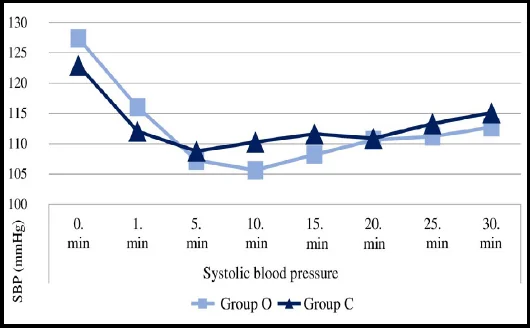
Comparison of systolic blood pressure values between the groups.

**Fig.3:**
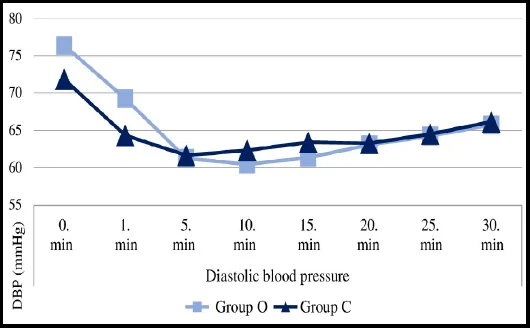
Comparison of diastolic blood pressure values between the groups.

**Fig.4:**
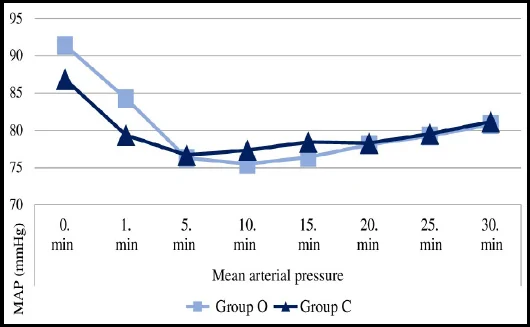
Comparison of mean arterial pressure values between the groups.

The need for additional uterotonic agents during the 0–5 minute period was significantly lower in Group-C (2.0%) compared to Group-O (12.0%) (p<0.05). There were no significant differences between Group-O and Group-C in additional uterotonic requirements during the 5–10, 10–15, 15–20, 20–25, and 25–30 minute intervals (p>0.05). However, the total uterotonic drug requirement throughout the operation was statistically significantly lower in Group-C (10.0%) than in Group-O (26.0%) (p<0.05) (Table-II)

**Table-II:** Evaluation of additional uterotonic requirements between the groups.

		Group-O (n:50)		Group-C (n:50)		p-value	
		n	%	n	%		
***Additional uterotonic agents***							
0 -5. min	(-)	44	88.0	49	98	0.050	^X²^
	(+)	6	12.0	1	2.0		
5-10. min	(-)	49	98.0	46	92.0	0.169	^X²^
	(+)	1	2.0	4	8.0		
10-15. min	(-)	49	98.0	50	100.0	1.000	^X²^
	(+)	1	2.0	0	0.0		
15-20. min	(-)	47	94.0	50	100.0	0.242	^X²^
	(+)	3	6.0	0	0.0		
20-25. min	(-)	49	98.0	50	100.0	1.000	^X²^
	(+)	1	2.0	0	0.0		
25-30. min	(-)	49	98.0	50	100.0	1.000	^X²^
	(+)	1	2.0	0	0.0		
Total (0-30. min)	(-)	37	74.0	45	90.0	0.035	^X²^
	(+)	13	26.0	5	10.0		

^X²^ Chi-square test (Fischer test), min: minute.

There was no significant difference in uterine tone between Group-O and Group-C at one minute (p>0.05). At five minutes, uterine tone was significantly higher in Group-C compared to Group-O (p<0.05). However, at 10 minutes, uterine tone was significantly lower in Group-C than in Group-O (p<0.05) (Table-III). Since no significant differences were found between Group-O and Group-C in peripheral oxygen saturation or mean heart rate values at any measurement time, these data were not presented graphically.

**Table-III:** Evaluation of uterine tone between the groups.

	Group-O (n:50)				Group-C (n:50)				p-value	
	Mean±SD			Median	Mean±SD			Median		
Uterine Tone										
1 minutes	5.5	±	2.1	5.5	6.3	±	1.2	6.0	0.061	^m^
5 minutes	7.3	±	1.5	7.0	8.0	±	0.9	8.0	***0.016***	^m^
10 minutes	8.8	±	1.0	9.0	8.3	±	1.2	9.0	***0.034***	^m^

^m^ Mann-whitney u test, min: minute.

## DISCUSSION

The main findings of this prospective, randomized, double-blind study demonstrate that carbetocin is associated with significantly reduced intraoperative blood loss, improved early uterine tone, and a lower requirement for additional uterotonic agents compared with oxytocin during elective cesarean delivery, while maintaining comparable intraoperative hemodynamic stability.

Although both agents showed similar effects on heart rate, blood pressure, and oxygen saturation, carbetocin provided superior uterine contraction in the early postoperative period, which likely contributed to reduced bleeding and a diminished need for supplementary uterotonics. The higher uterine tone observed at five minutes in the carbetocin group appears clinically relevant, as the early postpartum period is critical for preventing uterine atony–related hemorrhage.

These findings are consistent with previous studies reporting improved uterine tone and a reduced need for additional uterotonics with carbetocin. Borutto et al. evaluated the efficacy of a single intravenous bolus of 100 μg carbetocin compared with a two-hour infusion of oxytocin in patients undergoing cesarean delivery. They reported that, despite no significant difference in mean blood loss between the groups, the requirement for uterine massage and additional uterotonic agents was significantly lower in the carbetocin group.^10^ Notably, supplemental uterotonics were required more frequently in the oxytocin group, suggesting that uterine massage alone was insufficient to maintain optimal uterine tone. Similarly, Larciprete et al. reported no additional uterotonic requirements in the carbetocin group among high-risk patients, supporting its sustained uterotonic efficacy.^11^ More recently, a systematic review and meta-analysis by Maged et al. demonstrated that carbetocin was associated with reduced blood loss, lower transfusion requirements, and a decreased need for additional uterotonic agents compared with oxytocin following cesarean delivery.^12^ Likewise, Bonus et al. reported that carbetocin significantly reduced the requirement for additional uterotonic agents and blood transfusion compared with oxytocin in women undergoing cesarean delivery.^13^ Consistent with these findings, our study demonstrated a higher requirement for additional uterotonics in the oxytocin group (26%) compared with the carbetocin group (10%).

An interesting finding of the present study was that uterine tone scores were higher in the carbetocin group at five minutes, whereas higher scores were observed in the oxytocin group at 10 minutes. One possible explanation for this finding is the more frequent use of additional uterotonic agents in the oxytocin group during the early intraoperative period, particularly within the first five minutes following placental delivery. Therefore, the higher uterine tone scores observed at 10 minutes may have been influenced, at least in part, by supplemental uterotonic administration. Nevertheless, the oxytocin group demonstrated a significantly greater overall requirement for additional uterotonic agents and higher intraoperative blood loss, suggesting that the overall uterotonic efficacy of carbetocin remained favorable.

In contrast, Rabow et al. reported no significant differences in uterine tone or blood loss between oxytocin and carbetocin.^14^ Such discrepancies may be attributed to differences in study design, duration of observation, patient populations, methods used for uterine tone assessment, and drug administration protocols. Regarding hemodynamic outcomes, Bahr et al. compared the effects of oxytocin and carbetocin in preeclamptic patients undergoing elective cesarean delivery under spinal anesthesia.^15^ They reported greater increases in heart rate and more pronounced reductions in blood pressure and oxygen saturation in the oxytocin group, along with a higher incidence of postpartum hemorrhage and additional uterotonic use. More recently, Delavallade et al. demonstrated that carbetocin was more effective than oxytocin in preventing postpartum bleeding among women at high risk of hemorrhage undergoing cesarean delivery.^16^ Although differences in patient characteristics and anesthetic techniques limit direct comparison with our study population, these findings are broadly consistent with our results, which demonstrated lower intraoperative blood loss and reduced additional uterotonic requirements in the carbetocin group. Collectively, these studies further support the favorable efficacy profile of carbetocin across different cesarean delivery populations.

Recent evidence further supports the hemodynamic safety of carbetocin. Refaat et al. compared carbetocin and oxytocin in women with stenotic valvular heart disease undergoing cesarean delivery and reported more stable hemodynamic parameters with carbetocin while maintaining adequate uterine contraction.^17^ Although the study population differed from that of the present study, these findings further support the favorable hemodynamic profile of carbetocin observed in our patients.

Large-scale data further support the hemodynamic safety of carbetocin. Moertl et al. examined hemodynamic parameters in more than 2,500 patients undergoing cesarean delivery under neuraxial anesthesia and reported similar hemodynamic responses between carbetocin and oxytocin; however, repeated oxytocin dosing was associated with delayed hemodynamic recovery.**18** These findings highlight the potential benefit of a single-dose uterotonic strategy, as employed in our study, to minimize cumulative cardiovascular effects during cesarean delivery.

From a broader cardiovascular perspective, Svanström et al. demonstrated that oxytocin administration was associated with significant tachycardia, hypotension, and transient myocardial ischemic changes, whereas methylergometrine primarily increased blood pressure without significant cardiac effects.**19** Given the hypertensive profile of methylergometrine and its limited role as a first-line uterotonic, these findings emphasize the need for safer alternatives. In this context, carbetocin may offer an advantage by providing effective uterine contraction while maintaining hemodynamic stability, particularly in patients with increased cardiovascular risk.

Overall, both agents were well tolerated in the present study. Although a transient decrease in mean arterial pressure was observed during the first minute in the carbetocin group, this effect was short-lived and clinically insignificant. Previous studies have reported associations between oxytocin administration and tachycardia, hypotension, and myocardial ischemia, particularly in susceptible patients.**20** Taken together, these findings support the use of carbetocin as a uterotonic agent with a favorable hemodynamic profile.

### Strengths:

The strengths of this study include its randomized double-blind design, standardized anesthesia and uterotonic administration protocols, and comprehensive assessment of uterine tone and intraoperative hemodynamic parameters.

### Limitations:

The study was conducted at a single center and included only patients undergoing elective cesarean delivery under general anesthesia, which may limit the generalizability of the findings. In addition, long-term maternal and neonatal outcomes were not assessed. Another limitation of the present study is that no formal cost-effectiveness analysis was performed. Although carbetocin was associated with reduced intraoperative blood loss and a lower requirement for additional uterotonic agents, its higher acquisition cost compared with oxytocin may influence its routine clinical use.

Further multicenter studies involving diverse anesthesia techniques, high-risk patient populations, and long-term maternal and neonatal follow-up are warranted to better define the optimal role of carbetocin in obstetric practice.

## CONCLUSION

Both oxytocin and carbetocin were found to be safe and effective for promoting uterine contraction during elective cesarean delivery, with comparable intraoperative hemodynamic profiles. However, carbetocin was associated with reduced intraoperative blood loss, improved early uterine tone, and a lower requirement for additional uterotonic agents. Its single-dose administration and favorable safety profile, together with its superior performance in reducing intraoperative blood loss and the need for additional uterotonic agents, support the preferential use of carbetocin in clinical practice.

### Recommendations:

Future studies incorporating pharmacoeconomic evaluations are needed to better determine the overall cost-effectiveness of carbetocin in cesarean delivery.
